# Assessing the Performance of Thin-Film Nanofiltration Membranes with Embedded Montmorillonites

**DOI:** 10.3390/membranes10050079

**Published:** 2020-04-26

**Authors:** Micah Belle Marie Yap Ang, Amira Beatriz Gaces Deang, Ruth R. Aquino, Blessie A. Basilia, Shu-Hsien Huang, Kueir-Rarn Lee, Juin-Yih Lai

**Affiliations:** 1 R&D Center for Membrane Technology and Department of Chemical Engineering, Chung Yuan Christian University, Taoyuan 32023, Taiwan; 2 School of Chemical, Biological, and Materials Engineering and Sciences, Mapúa University, Manila 1002, Philippines; 3 Industrial Technology Development Institute, Department of Science and Technology, DOST Compound, Taguig City 1631, Philippines; 4 Department of Chemical and Materials Engineering, National Ilan University, Yilan 26047, Taiwan; 5 Applied Research Center for Thin-Film Metallic Glass, National Taiwan University of Science and Technology, Taipei 10607, Taiwan; 6 Graduate Institute of Applied Science and Technology, National Taiwan University of Science and Technology, Taipei 10607, Taiwan

**Keywords:** montmorillonite, polyamide, thin-film nanocomposite, membrane separation, nanofiltration

## Abstract

In this study, the basal spacing of montmorillonite (MMT) was modified through ion exchange. Two kinds of MMT were used: sodium-modified MMT (Na-MMT) and organo-modified MMT (O-MMT). These two particles were incorporated separately into the thin-film nanocomposite polyamide membrane through the interfacial polymerization of piperazine and trimesoyl chloride in n-hexane. The membrane with O-MMT (TFN_O-MMT_) has a more hydrophilic surface compared to that of membrane with Na-MMT (TFN_Na-MMT_). When various types of MMT were dispersed in the n-hexane solution with trimesoyl chloride (TMC), O-MMT was well-dispersed than Na-MMT. The poor dispersion of Na-MMT in n-hexane led to the aggregation of Na-MMT on the surface of TFN_Na-MMT_. TFN_O-MMT_ displayed a uniform distribution of O-MMT on the surface, because O-MMT was well-dispersed in n-hexane. In comparison with the pristine and TFN_Na-MMT_ membranes, TFN_O-MMT_ delivered the highest pure water flux of 53.15 ± 3.30 L∙m^−2^∙h^−1^ at 6 bar, while its salt rejection for divalent ions remained at 95%–99%. Furthermore, it had stable performance in wide operating condition, and it exhibited a magnificent antifouling property. Therefore, a suitable type of MMT could lead to high separation efficiency.

## 1. Introduction

Treatment of industrial wastewater is vital before its discharge to rivers or soil land. The traditional treatment methods are screening, flotation, coagulation, and chlorination [1,2]. These methods are now at a disadvantage for consuming a lot of energy and requiring large working space to assemble. Conventional separation technique like membrane separation demonstrates its economical way of treatment [3,4]. One type of membrane separation is pressure-driven membrane filtration. Under this process are the following methods: microfiltration, ultrafiltration, nanofiltration (NF), and reverse osmosis (RO). These processes are often combined, depending on the type of wastewater [5,6,7]. Among these, NF is widely used for its applicability to various industries, such as water purification and desalination [8,9], food and beverages [7,10,11], semiconductor [12,13], petroleum [14,15], pharmaceutical and biotechnology [16,17] and the textiles industry [18,19].

Several methods can be used to fabricate NF membranes. These are the phase-inversion process, polymer coating (dip coating, spin coating, or solution casting), layer-by-layer self-assembly, grafting, and interfacial polymerization [20,21]. Interfacial polymerization remains the preferred method, because the membranes formed during this process exhibited high permeability and selectivity. Furthermore, the polymerization reaction remains fast and easy to scale up compared to other methods. At present, there is a need to enhance the efficiency of the thin-film composite (TFC) membranes to cope with the fast-paced industrialization around the globe that results in an increase in the demand for clean water.

The membrane produced by interfacial polymerization is widely known as the thin-film composite (TFC) membrane. The TFC membrane is composed of a thin-film polyamide that forms on top of the polymer support. The reaction of interfacial polymerization occurs in two immiscible phases; commonly with water and organic solvent such as n-hexane, toluene, cyclohexane [22]. To increase the performance of the membranes, the following methods are used: modification of supporting layer [23,24], varying types of monomers [25,26], inclusion of another reactant in aqueous or organic phase [27,28], or the introduction of nanoparticles in the reaction [29,30]. Introducing inorganic nanoparticles enhances membrane performance and antifouling property without sacrificing salt rejections. With the rapid advancement of nanotechnology, researchers focus on developing nanoparticles to improve the TFC membranes. These nanoparticles are silica [29,31], silver [30,32], graphene or graphene oxide [33,34], zeolites [35,36], nanoclays [37,38,39,40,41,42,43], quantum dots [44,45], and carbon nanotubes [46,47]. TFC membranes with embedded nanoparticles are called thin-film nanocomposite (TFN) membranes [48,49]. Of all the nanoparticles, nanoclays are the most environmentally friendly and abundant. It is also preferable for its water purification capability, that can enhance the stability of the polymers in terms of structural, mechanical, and thermal [50,51]. Moreover, it requires a small amount of clay to boost the performance of the membrane [52].

Clays are naturally hydrophilic, which makes it favorable to embed in the polymer matrix with the improved wettability of the polymer. On top of this, they have a high adsorption capacity and surface area. They are small and exhibit a unique chemical composition with micro-porosity and a layered structure. They are divided into 5 different groups—kaolinite, montmorillonite (MMT), illite, and chlorite groups [53]. Among them, the MMT layers expand most, because their intermolecular spaces can be easily penetrated by water. In the year 1890, in the US, bentonite was discovered to contain MMTs [54]. Since then, bulk of MMTs are sourced from bentonite rocks. MMT has a 2-D layered structure, with each layer made of two silica tetrahedral sheets, with a central alumina octahedral sheet. The layers are intertwined oxygen atoms and exchangeable cations such as calcium, magnesium, sodium, and water molecules [55]. MMTs also have ions that may undergo ionic substitution, capable of producing useful new materials for various applications [53]. For the membrane applications, several scholars [37,38,39,40,41,42,43] attempted to embed different types of nanoclay in a polyamide matrix. Recently, Li et al. [37], modified a synthetic nanoclay–laponite by functionalizing it with different metal ions. They improved both the flux and antifouling properties of the polyamide membrane prepared using m-phenylenediamine (MPD), and trimesoyl chloride (TMC). Zhao et al. [38] used PEG 200 to assist in the dispersion of laptonite in an aqueous MPD solution. In their study, the flux of TFN membrane increased while maintaining NaCl rejection. Kadhom and Deng [39] prepared nano bentonite through the solvothermal method. At low bentonite concentration, there was an improvement in the flux and salt rejection of the RO membrane. Maalige et al. [40] incorporated a sulfonated bentonite in a polyamide membrane. Sulfonated bentonite altered the alignment of the polyamide chain; and enhanced the hydrophilicity and antifouling property of the membrane. Tajuddin et al. [41,42] used an anionic clay-layered double hydroxide in fabricating the NF membrane from the reaction of piperazine (PIP) and TMC. They also improved both water flux and the antifouling property of the membrane. Dong et al. [43] compared the influence of anionic and cationic clays when embedded in polyamide membranes. The anionic clay was represented by a double layered hydroxide, while the cationic clay was represented by MMT. The particles were dispersed in a TMC solution. Both particles changed the property of the RO membranes. On the other hand, the membrane with MMT showed higher RO performance than that of the membrane with double layered hydroxide nanoparticles.

The majority of the studies embedded the clay in the polyamide membrane made of MPD and TMC monomer for RO. In NF, PIP is the commonly used monomer. Polyamide made from PIP and TMC do not possess the same membrane properties with a polyamide prepared from MPD and TMC. Because MPD has primary amines and benzene group, whereas PIP has secondary amines and no benzene group, they produce different cross-linking degree when they react with TMC. Our study aimed to improve the separation efficiency of the NF membrane using two modified MMT and compare their effect with the property of the TFN membrane. These two types of MMT particles were sodium- and surfactant- intercalated MMT. Adding MMT to the polyamide layer creates a water pathway made from the interface between MMT and polyamide. These water pathways improved the separation efficiency. Its dispersion in different solvents is easy to control through the functionalization of monomers by ion exchange. Furthermore, because MMT is abundant in the environment, it also reduces the preparation cost of the TFN membranes. Following our previous work [29], that showed the economical side of putting the particles in an organic phase, the MMTs were dispersed in TMC in a n-hexane solution. The two MMTs exhibited different dispersion properties depending on the element or compound that is intercalated in the nanosheet of the MMT. This factor was studied through membrane characterization and a performance test.

## 2. Materials and Methods

### 2.1. Materials

Philippine MMT was provided by Material Science Division, Industrial Technology Development Institute of the Department of Science and Technology, Taguig City, Philippines. Its property was similar to the previous work [56]. Sodium carbonate, manufactured by Ajax Chemicals Ltd., Sydney, Australia, was used to improve the basal spacing of Philippine MMT. Dialkyldimethyl ammonium chloride (DDAC) was a product of Hoechst Altiengesellschaft, Frankfurt, Federal Republic of Germany. Polysulfone (PSf) pellets (UDEL P-3500) and a nonwoven polyester, as a raw material for the support layer, were obtained from Amoco Performance Product, Ridgefield, CT, USA, Ahlstrom, Helsinki, Finland, respectively. N-methyl-2-pyrrolidone (NMP), as a solvent of PSf, was acquired from Tedia Company, Inc. (Fairfield, OH, USA). Polyethylene glycol (PEG) 20k (as additive ng PSf support) and PIP (as diamine monomer) were purchased from Alfa Aesar (Heysham, Lacashire, England). TMC (as an organic monomer) was supplied by Tokyo Chemical Industry Co. Ltd. (Tokyo, Japan). n-Hexane was from Echo Chemical Co. Ltd. (Miaoli, Taiwan). Bovine serum albumin (BSA) and phosphate buffer saline were procured at UniRegion Bio-Tech (USA). The following solutes were bought from Sigma-Aldrich (Saint Louis, MO, USA): Na_2_SO_4_, MgCl_2_, MgSO_4_, and NaCl. Distilled water was produced in the laboratory using the Lotun ultrapure water system (Lotun Technic Co. Ltd., New Taipei, Taiwan).

### 2.2. Synthesis of Philippine Sodium-montmorillonite and Organo-montmorillonite

In a beaker, 100 g of MMTs was dehydrated at 100 °C. Afterwards, it was mixed with 1000 mL water and 3 g of sodium carbonate to form a paste texture. The paste was mixed for 30-min at room temperature. Once dehydrated, the MMTs were pulverized for 15 min. Then, 375 mL of water was added to form the mixture into a paste for the second time. Lastly, it was dried at 100 °C and pulverized using a mechanical grinder. MMTs were sieved with a 200-mesh. The particles that passed through 200-mesh were used in the study. The cationic exchange capacity of Na-MMT is 84.2, which is according to the BaCl_2_/MgSO_4_ method [57].

The functionalization of the organic material on Na-MMT was based on the work of Favre and Lagaly [58]. DDAC was used as organic material to modify the MMTs. The amount of DDAC was 1.5 times of CEC of Na-MMT. On one blunger, 100 g of Na-MMT was mixed with DDAC for 30 min and it was transferred to a stoppered glass container to react with DDAC and Na-MMT for 72-h at 70 °C. Then, it was washed several times until the amount of C and N in the particles were constant, which was determined by a CNS-2000 Elemental Analyzer (LECO Corporation, Minneapolis, MN, USA). The obtained organically modified MMTs were called organo-modified MMT (O-MMT). MMTs were dried at 80 °C, then were pulverized, and were sieved with a 200-mesh. Afterwards, they were dried again at 70 °C for 24-h and were then transferred to a vacuum desiccator.

### 2.3. Preparation of Thin-film Nanocomposite Membranes

The PSf support was similar to our previous work [59]. Inside a mechanical mixer, a 16 wt % PSf/NMP solution with a total volume of 4 L was dissolved for 24-h. Then, it was transferred to a 5 L bottle and kept in the oven at 30 °C. After 24-h of degassing, the PSf solution was poured into a continuous casting machine that was equipped with a non-woven polyester—PSf was precipitated on top of the non-woven. This PSf support was transferred to a water bath and washed for a day, before it was stored in 1 wt % sodium bisulfite.

**Figure 1:**
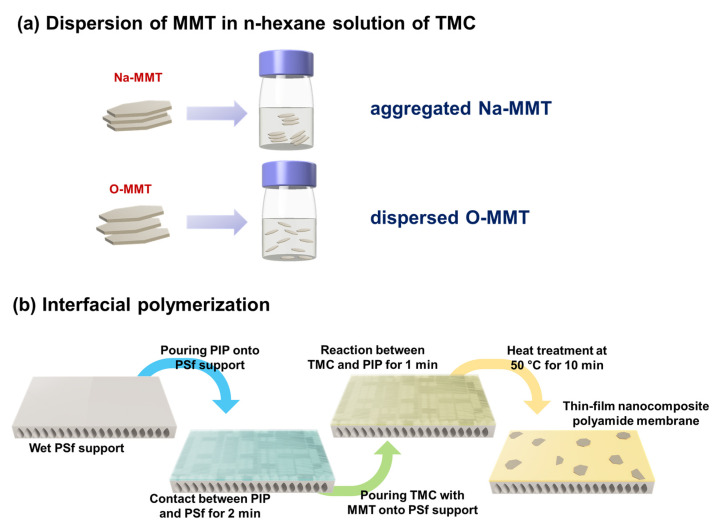
Schematic diagram of membrane preparation.

Figure 1 illustrates the preparation of the TFN membranes. Two types of MMTs were dispersed in the n-hexane solution of TMC. The material that was intercalated in the MMT affects the behavior of the clay in the oil solution [60]. Intercalating DDA^+^ to MMT would result in a better dispersion of MMT in the n-hexane solution than intercalating Na^+^. In Figure S1 (Supplementary Materials) are the photographs of the Na-MMT and O-MMT in n-hexane. The amount of the particle was fixed at 0.05 g O-MMT/50 mL n-hexane (low concentration) and 0.5 g of MMT/15 mL n-hexane (high concentration). Dispersing MMTs at low concentration cannot differentiate. On the other hand, at high concentration, Na-MMT settles immediately for less than 30 s, whereas O-MMT was still well-dispersed.

In the next step, 361 cm^2^ of PSf support was cut and washed three times with distilled water. Then, it was clamped to a metal holder and followed by pouring a 0.35 wt % aqueous PIP solution on the membrane surface. After 2 min, the solution was removed and disposed of in a waste bottle. The excess droplets on the surface were removed using an airgun at a pressure of 1 bar. Afterwards, the saturated membrane of PIP was again clamped on a metal plate. Then, 0.2 wt % TMC in n-hexane was poured on the PSf surface, and after a contact with the TMC solution, a thin-film polyamide layer formed in 1 min. The membrane was heat-treated at 50 °C for 10 min, then washed three times using distilled water. For the membranes containing MMT, 0.05 to 0.75 g of MMTs/g of TMC were added to a TMC in the n-hexane solution.

The membrane without MMTs was represented as TFC, whereas the membranes with MMTs were denoted as TFN_X_, where X stands for the Na-MMT or O-MMT.

### 2.4. Characterization of Montmorillonites and Composite Membranes

Attenuated total reflectance-Fourier transform infrared (ATR-FTIR) spectroscopy (Perkin Elmer Spectrum 100 FTIR Spectrometer, Waltham, MA, USA) was utilized to compare the chemical structure of MMTs and membranes. X-ray photoelectron spectroscopy (XPS, VG K-alpha ThermoFisher Scientific, Inc. Waltham, MA USA) determined the elemental composition of the membrane surface. Field emission scanning electron microscopy (FESEM, S-4800, Hitachi Co, Tokyo, Japan) was used to take the morphology of MMT and the membranes. The FESEM installed with energy dispersive X-Ray spectroscopy (EDX, Ultim^®^ Max, Oxford Instruments, High Wycombe, UK) obtained the Na content on the surface of the membrane. The surface roughness of the membranes was examined by an atomic force microscopy (AFM, NanoScope^®^ V, Bruker, Billerica, MA, USA). The MMTs were observed using transmission electron microscopy (TEM, JEOL JEM-2100, Tokyo, Japan). Thermodynamic gravimetric analysis (TGA, Q500, TA Instrument, USA) was employed to verify the degradation temperature of the MMTs. The crystallinity of the MMT and membranes were determined with X-ray diffraction (XRD, Model D8 Advance Eco, Bruker, Billerica, MA, USA). The charges of the MMTs were acquired using a dynamic light scattering instrument (DLS, Zeta Nano ZS, Malvern, UK), whereas the surface charge of the membrane was measured by a SurPASS Electrokinetic Analyzer (Anton Paar, New South Wales, Australia) under pH 3, 7, and 11. The membrane hydrophilicity was determined through an automatic interfacial tensiometer (PD-VP Model, Kyowa Interface Science Co. Ltd., Niiza-City, Saitama, Japan), with water droplet size of 5 µL during testing.

### 2.5. Evaluation of Nanofiltration Performance

Simultaneously, 4 pieces of membranes were tested in a crossflow device. Each membrane cell has an effective area (A) of 12.57 cm^2^. The membrane underwent a precompression at 6.5 bar for 1-h. After that, the pure water flux (J) was measured at 6 bar. The flux and salt rejection (R) were evaluated through the following equations:

(1) $$J=\frac{m}{\rho At}$$

(2) $$R=\frac{C_{f}-C_{i}}{C_{f}}\times 100\%$$

where *m* (kg) was the mass of permeate collected at time t (h), ρ was the water density (1 kg/L), and *C_f_* and *C_i_* were the concentrations of salt in feed (1000 ppm) and permeate, respectively. The salt concentration was measured using a Mettler Toledo SevenMulti (Schwerzenbach, Switzerland).

### 2.6. Evaluation of Antifouling Property and Stability of the Membrane

A crossflow filtration setup was used to evaluate the antifouling property of the membranes. The membrane was pre-pressurized at 6.5 bar for 1-h through supplying a distilled water. After 1-h, the pressure was decreased to 6 bar and the flux was measured every 10 min. (1) The feed was then replaced with a solution containing 0.1 g/L BSA, and 1000 ppm aqueous Na_2_SO_4_ solution. The pH was maintained at 7.4, through the addition of phosphate buffered saline solution. (2) The flux was measured every 10 min for 1-h. (3) Afterwards, this was washed by supplying distilled water for 15 min at 1 bar and another 15 min at 6 bar. (4) Then, the feed was swapped with fresh distilled water and the flux was measured three times every 10 min. Procedures (1) to (4) were repeated twice.

For the stability test, the membrane was precompressed for 1-h at 6.5 bar with distilled water. The feed was replaced with 1000 ppm Na_2_SO_4_, then flux and rejection were obtained at 6 bar. The operation was continued for 169-h. Flux and salt rejection were determined at random times.

## 3. Results and Discussion

### 3.1. Characterization of Montmorillonite

**Figure 2:**
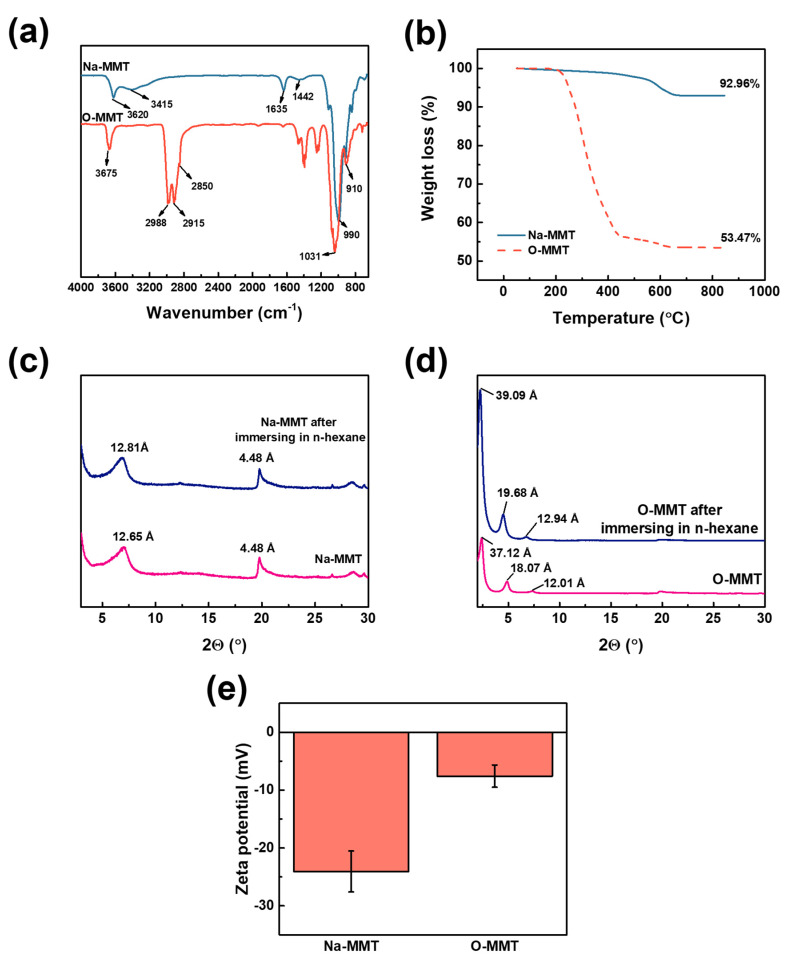
(**a**) ATR-FTIR spectra; (**b**) thermogravimetric analysis; (**c**,**d**) crystallinity; (**e**) zeta potential of the Na-MMT and organo-modified MMT (O-MMT).

Figure 2a indicates the ATR-FTIR of Na-MMT and O-MMT particles. The Na-MMT and the O-MMT had a peak at 3620 and 3675 cm^−1^, respectively. These peaks correspond to hydroxyl groups of MMT. However, only Na-MMT had a peak at 3415 cm^−1^, which was attributed to its bound water molecules. For O-MMT, the peak of water molecules cannot be observed because of the intercalation of DDA^+^ on the interlayer spaces of MMT. When Na-MMT and DDAC underwent an ion exchange, DDA^+^ replaced the Na^+^. The hydrophobic alkanes chains of DDA^+^ cannot catch water molecules. The Si-O-Si of the Na-MMT and O-MMT was located at 990 and 1031 cm^−1^, whereas the stretching vibration of Al-O-(OH)-Al was at 910 cm^−1^. The H-OH bond from water molecules that was retained in the MMT was positioned at 1635 cm^−1^. Carbonates in the MMT were located at 1460 cm^−1^. New peaks were appeared from O-MMT at 2850 and 2915 cm^−1^, which ascribed to the -CH_2_ and -CH_3_ stretching vibration of long alkyl chain of DDA^+^, respectively. Moreover, the peak at 2988 cm^−1^ was corresponded to the N-H stretching of amine salts [61,62,63,64].

The degradation temperature and percent residue of MMT were determined using thermogravimetric analysis (Figure 2b). The Na-MMT reduced its weight by 0.16% within the temperature range of 50 and 100 °C—this loss was from the adsorbed water. Between the temperature of 100—700 °C, 6.91% was lost because of the dehydroxylated of aluminosilicate layer. Hence, the total weight loss of Na-MMT was 7.04%. On the other hand, O-MMT had a total weight loss of 46.43%. The larger difference in weight loss of O-MMT than Na-MMT was because of the presence of the DDA^+^. Ahmad et al. [65] reported similar results. At 50 to 100°C, the O-MMT lost the bound water. Two elements decomposed between the temperature range of 100 and 700 °C; these were the amine salts of DDA^+^ and the dihydroxylation of aluminosilicate layer.

Figure 2c,d confirms the intercalation of Na^+^ and DDA^+^ in MMT. Inglethorpe et al. [57] obtained similar results; that the basal spacing of Na-MMT was 12.65 Å, under normal relative humidity. After swelling the Na-MMT in n-hexane solution, the d-spacing increased to 12.81 Å. Thus, Na-MMT was swollen by the n-hexane only. When some Na^+^ was replaced with DDA^+^, at 2Θ range from 2°–10°, three basal spacing emerged. These spacings had values of 37.12, 18.07 and 12.01 Å, indicating that the O-MMT was composed of paraffinic, mono-, and bi-layers, respectively [66,67]. Therefore, O-MMT had Na^+^ and DDA^+^ that was intercalated in the MMT nanosheets. When O-MMT was dispersed in n-hexane, the d-spacing enlarged at a greater degree. Spaces of the bilayers increased from 12.01 to 12.94 Å; monolayers transformed to pseudotrimolecular layers from 18.07 to 19.68 Å; and paraffinic layers also expanded from 37.12 to 39.09 Å. Figure 2e defines the zeta potential of the particles at pH 7. Na-MMT had a negatively charged surface of −24.07 ± 3.53 mV, because it is composed of an aluminosilicate that emits electronegativity. However, after intercalation of DDA^+^, the net charge of O-MMT particles became −7.60 ± 1.92 mV. This was because the DDA^+^ neutralized some part of the aluminosilicate. Therefore, intercalating DDA^+^ changed the net charge of MMTs into less negatively charged MMTs [68,69].

**Figure 3:**
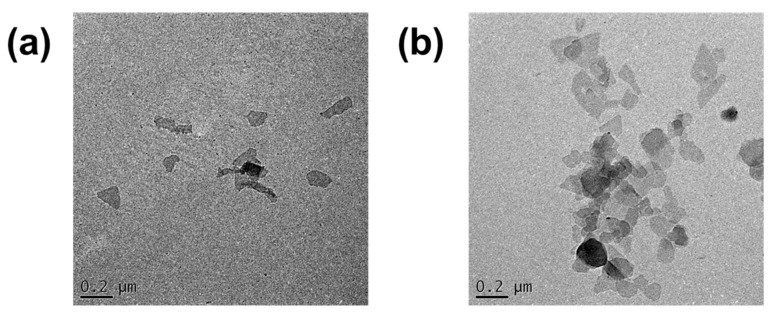
Particle morphology of (**a**) Na-MMT and (**b**) O-MMT.

Figure 3 reveals the TEM images of Na-MMT and O-MMT nanoparticles. The random stacking of MMT was visible. The distance between sheets was difficult to measure, because MMT consists of sheets that were folded at the edges. Table S1 (Supplementary Materials) enumerates the surface area, pore volume and pore size of the MMTs. Na-MMT and O-MMT had Brunauer–Emmett–Teller surface areas of 40.12 and 5.05 m^2^∙g^−1^, respectively. The pore size of Na-MMT was 129.23 Å, whereas O-MMT was 205.34 Å.

### 3.2. Characterization of the Membranes

#### 3.2.1. Surface Chemical Composition

**Figure 4:**
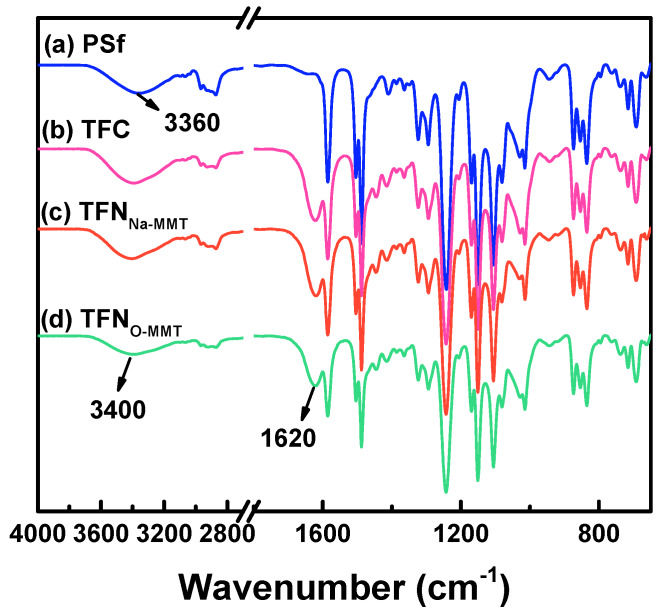
ATR-FTIR spectra of polysulfone (PSf), thin-film composite (TFC) and thin-film nanocomposite (TFN) membranes. Particle concentration = 0.05 g O-MMT/g TMC.

**Figure 5:**
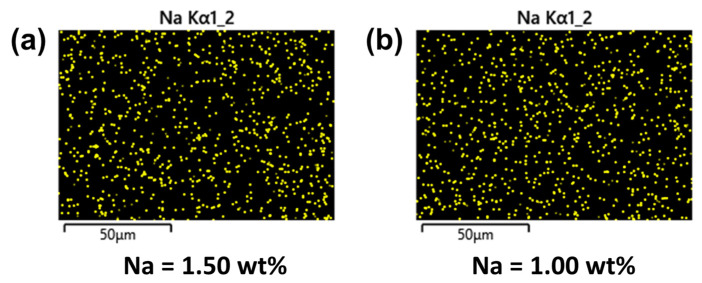
EDX mapping of Na element: (**a**) TFN_Na-MMT_; (**b**) TFN_O-MMT_. Particle concentration = 0.05 g O-MMT/g trimesoyl chloride (TMC).

Figure 4 indicates the chemical structure of PSf support, TFC, and TFN membranes. The peaks at 1587, 1504, and 1488 cm^−1^ were attributed to the aromatic rings of PSf support. The asymmetric and symmetric S = O stretching vibrations of PSf were located at 1327–1293 and 1178–1147 cm^−1^, respectively [70,71]. The PSf support had a peak at 3360 cm^−1^, which was attributed to the addition of PEG 20k into the PSf solution [72]. TFC and TFN membranes also had a broad peak at 3100–3650 cm^−1^, corresponding to O-H and N-H stretching of the carboxylic acid and secondary amine, respectively. The carboxylic acid was from the hydrolysis of TMC, formed during the washing of the membrane using water. The secondary amine was from the PIP monomer that was not reacted with the acyl chloride of TMC. A new peak was found at 1620 cm^−1^ for TFC and TFN membranes, assigned to the amide I of polyamide layer formed from the reaction of PIP and TMC [73,74,75]. The peak of MMT, however, cannot be seen, because of the overlapping spectra of PSf support and MMT. It was also possible that particles on the sample were too few to be detected. Therefore, XPS (Table 1) and EDX (Figure 5) scanned the elemental composition on the membrane surface.

Table 1 lists the elemental composition of TFC and TFN membranes. The TFC membrane only contained 70.3% C, 16.9% O and 12.9% N. By incorporating MMT into the polyamide layer, new elements were detected, such as Ca, Na, Si, Fe, Al, Mg, Ti, P, and K. These elements were common for MMT. TFN_O-MMT_ had higher N content than that of TFN_Na-MMT_, because the amine salt DDA^+^ was intercalated into O-MMT of TFN_O-MMT_. Figure 5 mapped the dispersion of the Na element on the TFN membrane surface, using EDX analysis. Because MMT also contains some Na_2_O on the nanosheet [76], it was used to monitor the dispersion of MMT on the membrane surface. Other elements could have been blocked by the X-ray in the EDX. Both TFN membranes showed a uniform distribution of the Na element of MMT. TFN_Na-MMT_ had more Na (1.5 wt %) on the surface, because the particle embedded here was modified by Na^+^. TFN_O-MMT_ had only 1.0 wt % Na, because Na^+^ of Na-MMT was replaced by the DDA^+^ of the surfactant. Therefore, the Na on the surface of TFN_O-MMT_ could be from the Na_2_O of the MMT and some Na^+^ that was not replaced by DDA^+^.

#### 3.2.2. Membrane Morphology and Structure

**Figure 6:**
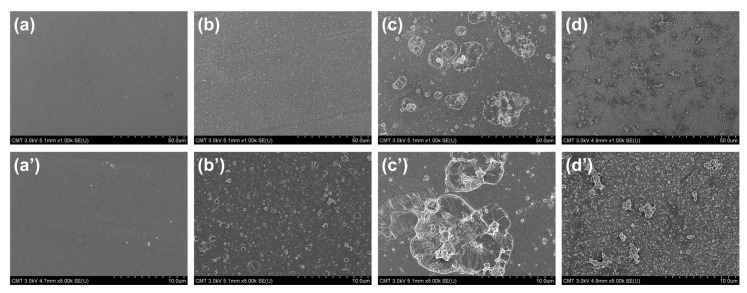
Surface field emission scanning electron microscopy (FESEM) images (magnification = ×1k and ×5k) of (**a**,**a’**) PSf, (**b**,**b’**) TFC, (**c**,**c’**) TFN_Na-MMT_ and (**d**,**d’**) TFN_O-MMT_. Particle concentration = 0.05 g O-MMT/g TMC.

Figure 6 presents the surface FESEM images of PSf support, TFC, and TFN membranes. The TFC membranes had nodules on the surface, which was a typical surface for a polyamide membrane prepared from PIP and TMC. When Na-MMT was embedded, the TFN_Na-MMT_ had a less uniform surface and the aggregation of Na-MMT on the surface was visible. The aggregation of Na-MMT on the polyamide surface was the result of poor dispersion of Na-MMT nanosheets in n-hexane. When Na-MMT was immersed with an oil solution, only a swelling of the nanosheet in the oil would occur [60]. This could result to numerous lamellar stackings of Na-MMT on the polyamide matrix. To improve the property of MMT, Na-MMT was modified through ion exchange using DDAC. DDAC is a surfactant with a hydrophobic tail and a hydrophilic head that would help the MMT to disperse better in the n-hexane solution. Compared to TFN_Na-MMT_, TFN_O-MMT_ had less aggregation of O-MMT on the surface, because O-MMT dispersed well in in-hexane solution. Thus, O-MMT distributed more uniformly into the polyamide structure after interfacial polymerization compared to Na-MMT nanoparticles.

**Figure 7:**
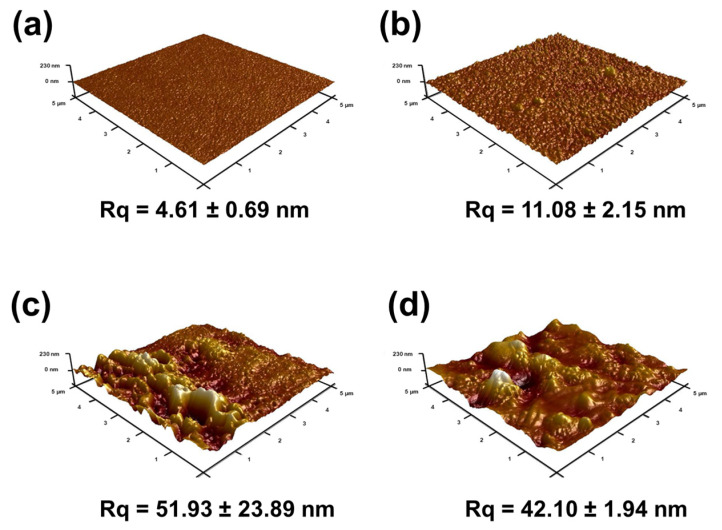
Three-dimensional atomic force microscopy (AFM) images of (**a**) PSf, (**b**) TFC, (**c**) TFN_Na-MMT_ and (**d**) TFN_O-MMT_. Particle concentration = 0.05 g O-MMT/g TMC.

The surface roughness also affected the membrane performance (Figure 7). AFM analysis had similar results with surface FESEM images (Figure 6) of the membranes. PSf support had the smoothest surface with a root mean square (Rq) of 4.61 ± 0.69 nm. When PIP and TMC reacted on top of the PSf to form a polyamide layer, the surface roughness increased to 11.08 ± 2.15 nm. This increase in surface roughness was because of the nodules on its surface (Figure 6b). Compared to TFN_O-MMT_ (42.1 ± 1.94 nm), TFN_Na-MMT_ had a higher surface roughness (51.96 ± 23.89 nm). This high surface roughness was from the aggregation of the Na-MMT on the polyamide matrix. The O-MMT was well-dispersed when transferred to the n-hexane solution compared to the Na-MMT, thus, the lamellar nanosheets of Na-MMT had a higher tendency to aggregate when embedded on polyamide layer. Nevertheless, both TFN membranes had a higher surface roughness than that of TFC. The advantage of a membrane with rougher surface is that there is a larger area for water to contact, therefore providing higher water flux. Similar to other works [42,77,78], embedding nanoparticles enhanced the surface roughness of the polyamide.

**Figure 8:**
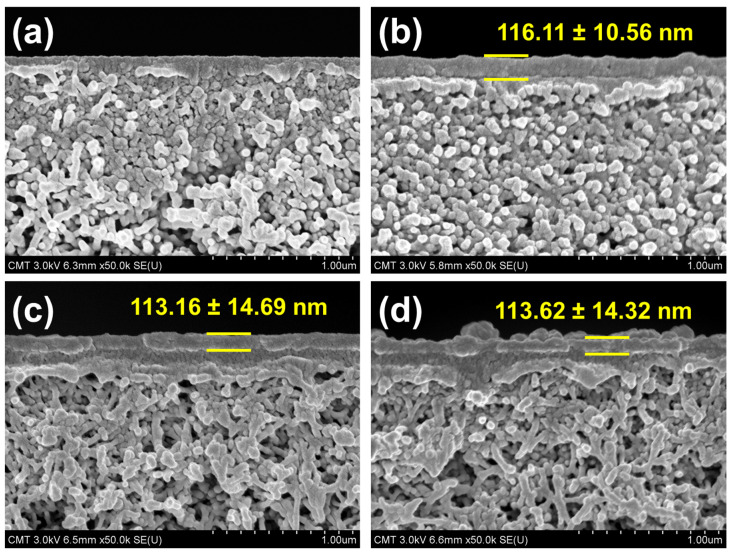
Cross-sectional FESEM images of (**a**) PSf, (**b**) TFC, (**c**) TFN_Na-MMT_ and (**d**) TFN_O-MMT_. Particle concentration = 0.05 g O-MMT/g TMC.

The thickness of the polyamide layer may be observed through the photo of cross-sectional FESEM images (Figure 8). When the polyamide layer was deposited on PSf surface, a thin layer of polyamide was formed, with an average thickness of about 116.11 ± 10.56 nm. However, incorporating either Na-MMT or O-MMT, TFN membrane had a thinner polyamide layer than that of TFC membrane, which was approximately 113 nm. The decrease in thickness of the polyamide may have transpired during interfacial polymerization, when MMT possibly prevented some TMC to react with PIP, therefore slowing down the reaction rate. Other works [59,77] also obtained similar findings.

#### 3.2.3. Crystallinity, Wettability and Surface Charge

**Figure 9:**
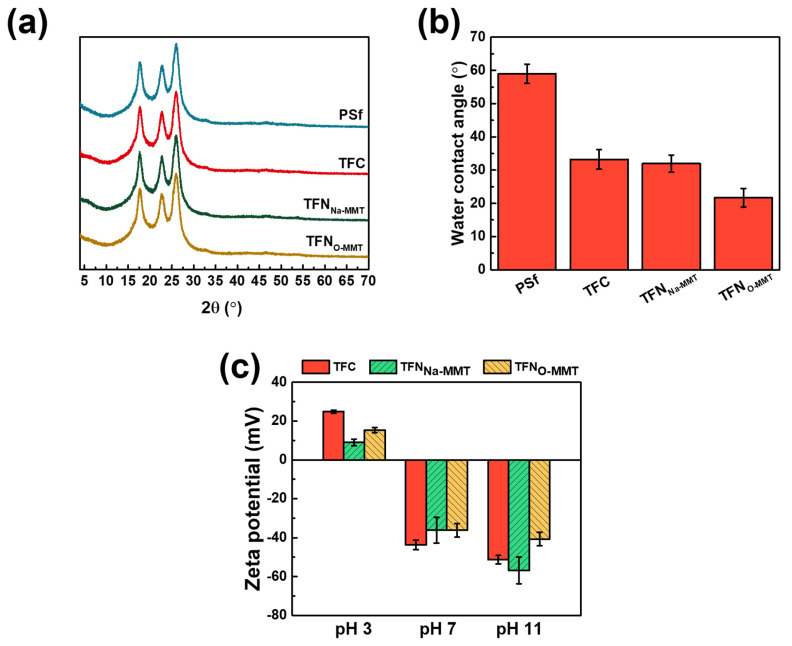
(**a**) XRD analysis; (**b**) water contact angle; and (**c**) zeta potential analysis of the membranes. Particle concentration = 0.05 g O-MMT/g TMC.

Figure 9a evaluates the crystallinity of the membrane. However, there was no difference on the XRD patterns of PSf, TFC, and TFN membranes. All the peaks were corresponded to the spectra of PSf support. Furthermore, no peaks of Na-MMT or O-MMT were observed on TFN membranes—indicating that the particles on the polyamide were exfoliated. The water contact angle of the membranes defines their hydrophilicity. Two factors can affect the water contact angle—the surface functional groups and the surface roughness. Figure 9b reveals that TFN_O-MMT_ had the lowest water contact angle, because of the hydrophilic nature of O-MMT. In addition, TFN_O-MMT_ had a rougher surface (Figure 7) than that of TFC, therefore providing a larger area for water to contact [79]. The water contact angle of the membranes was as follows: PSf = 58.98 ± 2.88°; TFC = 33.23 ± 2.96°; TFN_Na-MMT_ = 31.95 ± 2.59°; TFN_O-MMT_ = 21.69 ± 2.79°.

Figure 9c evaluates the surface zeta potential of the TFC and TFN membranes at pH 3, 7, and 11. At pH 3, all membranes had a positively charged surface, because their amine group protonated to NH^+^ thus giving a surface with net positive charge. The order from high to low of the zeta potentials at pH 3 were as follows: TFC (24.86 ± 0.78 mV) > TFN_O-MMT_ (15.30 ± 1.34 mV) > TFN_Na-MMT_ (8.94 ± 1.75 mV). During interfacial polymerization of the TFC membrane, no particle hindered the reaction of PIP and TMC, thus producing more amide groups on the membrane surface and less carboxylic acid group from the hydrolysis of TMC, resulting in a more positively charged surface. TFN_O-MMT_ had a more positively charged surface than that of TFN_Na-MMT_ at pH 3. Because TFN_O-MMT_ had DDA^+^ intercalated in O-MMT lamellar sheets, its amine salt also protonated, probably producing a more positively charged membrane than that of TFN_Na-MMT_. At pH 7 and 11, all membranes had a negatively charged surface. For the TFC membrane, only carboxylic acid groups deprotonated to COO^−^, whereas TFN membranes had aluminosilicates that also deprotonated. Therefore, TFN_Na-MMT_ and TFN_O-MMT_ had similar zeta potential of −36 mV, whereas TFC had −43 mV. The net charge of TFC membrane at pH 7 was more negative, because there was no particle on its surface that would contribute positively charged molecules. However, TFN_Na-MMT_ exhibited the most negative at a very basic condition (pH 11), because Na-MMT was abundant with an aluminosilicate that also deprotonates. For TFN_O-MMT_, it had DDA^+^ that was intercalated in the O-MMT―produces less negative on the net charge of the membrane at pH 11. Nevertheless, the zeta potential of the membranes at pH 3 to 11 was similar to a negatively charged NF membrane.

### 3.3. Nanofiltration Performance

**Figure 10:**
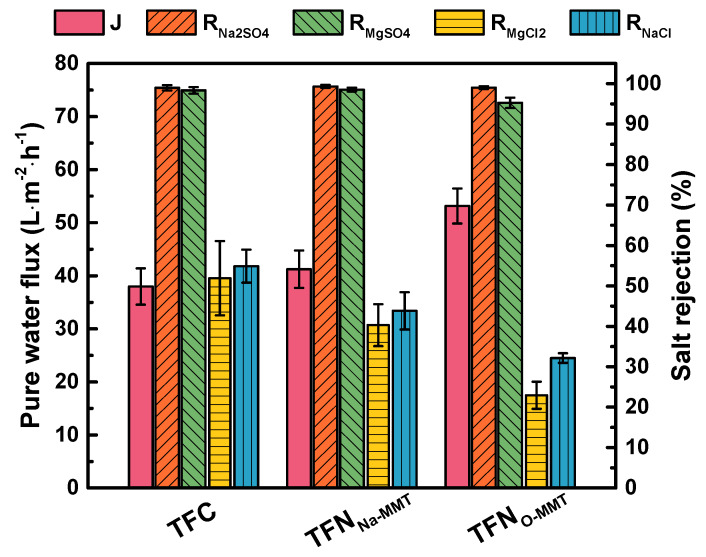
Nanofiltration performance of composite membranes. Feed = 1000 ppm salt solution; Operating condition: pH = 7, 6 bar, 30 °C. Particle concentration = 0.05 g O-MMT/g TMC.

Figure 10 compares the performance of TFC and TFN membranes using four different salts. TFN_O-MMT_ had the highest pure water flux of 53.15 ± 3.30 L·m^−2^·h^−1^because it had the most hydrophilic surface. Compared to the TFC membrane (37.98 ± 3.41 L·m^−2^·h^−1^), TFN_Na-MMT_ had a higher pure water flux (41.24 ± 3.53 L·m^−2^·h^−1^), because it had a rougher surface, where more water could contact on its surface, resulting in a higher flux. The salt rejection of all membranes follows the typically negatively charged membranes: Na_2_SO_4_ ≅ MgSO_4_ > MgCl_2_ ≅ NaCl. The ions had the following hydrated radii in decreasing order: Mg^2+^ (0.43 nm) > SO_4_^2−^ (0.38 nm) > Na^+^ (0.36 nm) > Cl^−^ (0.33 nm) [80]. The rejection for Na_2_SO_4_ and MgSO_4_ of all membranes was approximately between 95% and 99%. Na_2_SO_4_ and MgSO_4_ contain divalent anions. According to Donnan exclusion theory [81], a negatively charged membrane surface rejects divalent anions efficiently. However, after incorporating Na-MMT or O-MMT on the membrane, the MgCl_2_ and NaCl rejection of TFN membrane became relatively lower than that of TFC membrane. Because during interfacial polymerization, MMT interfered in the reaction of PIP and TMC, resulting in a less cross-linked structure of the polyamide. In addition, Na^+^ and Cl^−^ have a weaker electrical charge and a smaller hydrated radius compared with divalent ions. Thus, the rejection of NaCl for TFN membranes was lower than that of TFC. Nonetheless, the TFN_O-MMT_ membrane had a higher selectivity on the divalent salts over the monovalent salts.

**Figure 11:**
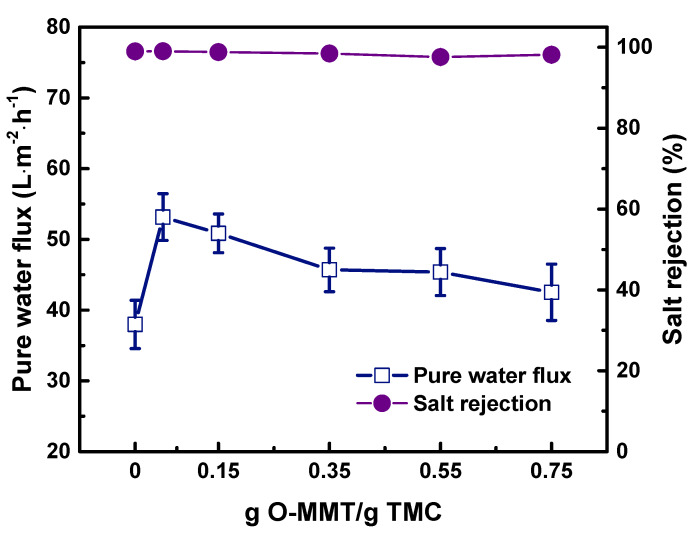
Nanofiltration performance of TFN_O-MMT_ membrane at varying weight ratio of O-MMT and TMC. Feed = 1000 ppm Na_2_SO_4_ solution; Operating condition: pH = 7, 6 bar, 30 °C.

Figure 11 plots the amount of O-MMT vs. NF performance. At 0.05 g O-MMT/g TMC, the TFN_O-MMT_ delivered the highest performance: pure water flux = 53.15 ± 3.30 L·m^−2^·h^−1^; rejection of Na_2_SO_4_ = 99.04 ± 0.35%. However, at above 0.05 g O-MMT/g TMC, the pure water flux began to decrease. This was because at a high concentration of O-MMT, it still act as a barrier for water to pass through, leading to an increase in mass transfer resistance and led to low water flux. Therefore, when the amount of O-MMT was increased from 0.05 to 0.75 g O-MMT/g TMC, the pure water flux simultaneously decreased from 53.15 ± 3.30 to 42.51 ± 3.98 L·m^−2^·h^−1^. Nevertheless, there was an improvement in membrane performance at the optimum concentration of O-MMT.

### 3.4. Operating Conditions

**Figure 12:**
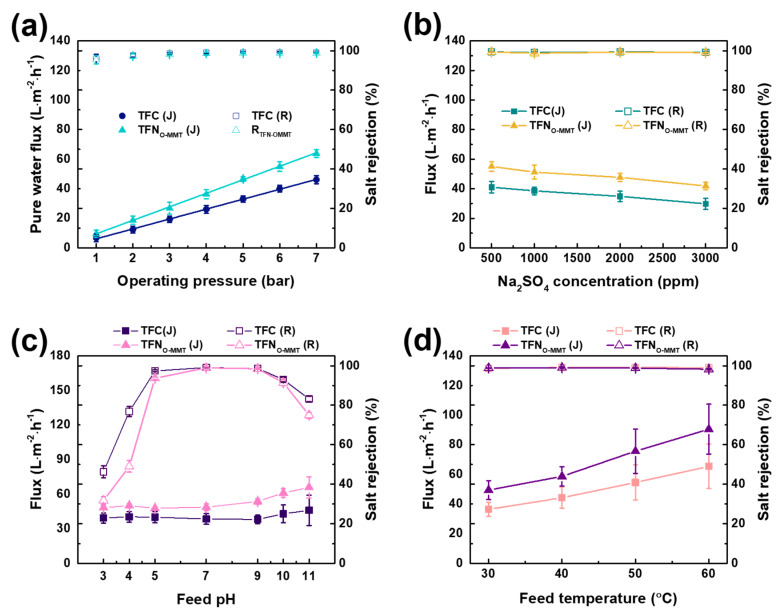
Nanofiltration performance of TFC and TFN_O-MMT_ at different operating conditions: (**a**) operating pressure; (**b**) Na_2_SO_4_ concentration; (**c**) feed pH; (**d**) feed temperature. Particle concentration = 0.05 g O-MMT/g TMC.

The testing conditions were varied to determine if the TFN_O-MMT_ is stable at a wide range operation. Figure 12a reports the performance of TFC and TFN_O-MMT_ membranes at different operating pressures from 1 to 7 bar. Both membranes displayed a linear increase in pure water flux. From 1 to 7 bar, pure water flux of TFC membrane increased from 6.71 ± 2.62 to 46.09 ± 2.70 L·m^−2^·h^−1^, whereas the flux of TFN_O-MMT_ rose from 9.45 ± 2.49 to 63.75 ± 2.69 L·m^−2^·h^−1^. Both membranes maintained the salt rejection up to 99%. These results showed that TFN_O-MMT_ still exhibited an advantage at a higher pressure, up to 7 bar.

The concentration of Na_2_SO_4_ in the feed varied from 500 to 3000 ppm (Figure 12b). At an increasing Na_2_SO_4_ concentration, the water flux of TFC and TFN_O-MMT_ decreased from 41.00 ± 3.79 to 29.81 ± 3.67, and 55.06 ± 3.17 to 41.86 ± 2.54 L·m^−2^·h^−1^, respectively. The decrease of flux was attributed to the high osmotic pressure at high salt concentration, causing a decrease in the driving force to push the water to the other side of the membrane [82]. Furthermore, with a high salt concentration, the salts gathered on the membrane surface and a phenomenon called concentration polarization occurred. This event gives another mass transfer resistance that prevents the water from passing through the membrane [83]. Nonetheless, the salt rejection of the TFC and TFN_O-MMT_ membrane was maintained at 99%. This shows that TFN_O-MMT_ membrane could also operate at a wide salt concentration.

The feed pH affects the surface charge of the membrane; hence, it is important to determine the performance of the membrane from pH 3 to 11 (Figure 12c). Increasing the pH from 3 to 5, the Na_2_SO_4_ rejection of TFC and TFN_O-MMT_ membranes was boosted from 46.24 ± 2.98 to 97.38 ± 0.48, and 31.74 ± 2.05 to 93.69 ± 0.64%, respectively. TFC and TFN_O-MMT_ had low rejection at pH below 5. At low pH, the amine groups of TFC and TFN_O-MMT_ membranes would protonate and give a more positively charged surface. This results in a higher chance of the SO_3_^2−^ being attracted to the membrane, leading to a higher chance of passing through the membrane. At pH 5 to 10, the salt rejection of the membrane was maintained at approximately 92% to 99%. At this pH, both membranes had a negatively charged surface because of deprotonation of the carboxyl groups and aluminosilicate groups in polyamide and O-MMT, respectively. According to Donnan exclusion, a negatively charged surface repels the anions (SO_3_^2−^) in a feed, resulting in high salt rejection. However, at pH 11, polyamide layer of TFC and TFN_O-MMT_ was already swelled because most of the carboxyl groups or metal oxides were deprotonated―a repulsion among them and creating larger free volume in the polyamide chain. Hence, the salt rejection of TFC and TFN_O-MMT_ at pH 11 declined to 83.18 ± 1.37 and 74.93 ± 1.55%, respectively. This displays the importance of the feed pH in achieving the optimum operating condition.

Figure 12d depicts the effect of feed temperature (30–60 °C) on the NF performance. TFC and TFN_O-MMT_ membranes had a stable salt rejection at low and high operating temperatures, which was approximately 99.00%. TFN_O-MMT_ had a higher flux than that of the TFC membrane for all range of temperature. With the increase of operating temperature from 30 to 60 °C, the flux of TFN_O-MMT_ increased from 49.36 ± 6.35 to 90.50 ± 16.83 L·m^−2^·h^−1^, whereas the flux of TFC also increased from 36.32 ± 4.73 to 65.32 ± 15.00 L·m^−2^·h^−1^. At a high temperature, the mobility of water moves faster in comparison to a low temperature, thereby enhancing the water flux. The results indicated that TFN_O-MMT_ was more advantageous than TFC membrane at a wide operating temperature.

### 3.5. Antifouling Property and Stability of the Membranes

**Figure 13:**
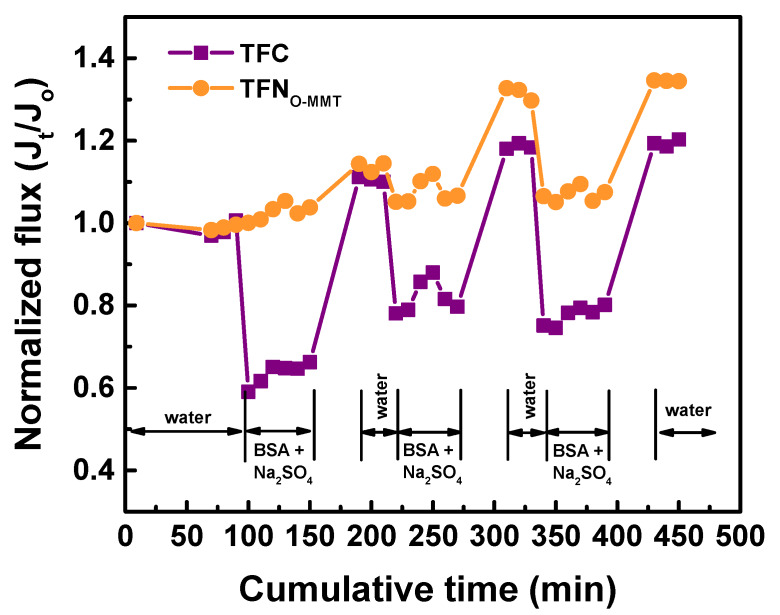
Antifouling property of TFC and TFN_O-MMT_. Feed = 100 ppm BSA + 1000 ppm Na_2_SO_4_; Operating condition: pH = 7.4, 6 bar, 30 °C. The normalized flux was calculated as the ratio of flux (J_t_) at time t over the initial flux (J_o_) measurement.

**Figure 14:**
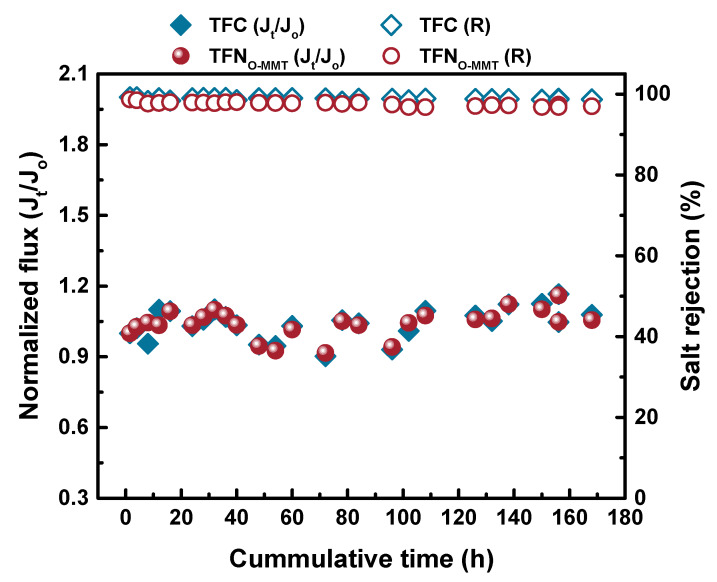
Stability test of TFC and TFN_O-MMT_ membrane for 168-h. Feed = 1000 ppm Na2SO4 solution; Operating condition: pH = 7, 6 bar, 30 °C. The normalized flux was calculated as the ratio of flux (J_t_) at time t over the initial flux (J_o_) measurement.

The antifouling property of the membranes should be improved to increase their lifespan. Figure 13 demonstrates the antifouling behavior of TFC and TFN_O-MMT_. The model foulant used was BSA. The fouling test was conducted for 3 cycles with the pH maintained at 7.4 using a phosphate-buffer saline solution. Similar to other works [84,85], reporting the normalized flux describes the antifouling capability of the membranes. For the first two cycles, the TFN_O-MMT_ membrane had a similar normalized flux when the feed was either pure water or BSA+Na_2_SO_4_ solution. After three cycles, the normalized flux of the TFN_O-MMT_ membrane increased to 1.34, whereas the TFC membrane had a normalized flux of 1.20. These results indicated that the TFN_O-MMT_ membrane had a better antifouling property. Other works [86,87] reported that when BSA containing phosphate buffers saline in contact with the membranes, it enlarged the membrane pores, resulting in an increase in flux. Figure 14 describes the stability of the TFC and TFN_O-MMT_. It was evident that the membrane remained stable for 168-h, even after O-MMT modified the polyamide layer. Therefore, the membrane was stable for long term use.

## 4. Conclusions

The behavior of the nanoparticles in dispersion medium is critical to obtain a high membrane performance. The two types of MMT behaved differently when dispersed to n-hexane in TMC solution. Intercalating DDA^+^ in MMT produced a better dispersed MMT in n-hexane than intercalating Na^+^. The surface of both MMTs became rough, resulting in an improvement in water flux and hydrophilicity. The TFN_O-MMT_ had a more uniform surface than TFN_Na-MMT_, because of the exfoliation of O-MMT in the n-hexane solution. The TFN_O-MMT_ also had a more hydrophilic surface in comparison to other membranes, because the O-MMT was more hydrophilic than that of Na-MMT. Compared to pristine and TFN_Na-MMT_, TFN_O-MMT_ delivered the highest NF performance at its optimum concentration. TFN_O-MMT_ was also stable on a wide operating condition and was suitable for long term use. Furthermore, the process of embedding O-MMT enhanced the antifouling property of the polyamide membrane.

## Figures and Tables

**Table 1:** Elemental composition of the membrane surface from XPS analysis.

	C (%)	O (%)	N (%)	Other Elements (%) ^a^
TFC	70.3	16.9	12.9	
TFN_NA-MMT_	74.8	14.5	7.64	3.02
TFN_O-MMT_	71.3	16.6	8.69	3.38

^a^ Ca, Na, Si, Fe, Al, Mg, Ti, P, K.

## Supplementary Materials

The following are available online at https://www.mdpi.com/2077-0375/10/5/79/s1, Figure S1: Dispersion of Na-MMT and O-MMT in n-hexane, Table S1: Surface area, total pore volume and pore size of MMTs.
