# Supplementary material for: Assessing the Performance of Thin-Film Nanofiltration Membranes with Embedded Montmorillonites
Source: Membranes (Basel). 2020 Apr 26;10(5):79. doi: 10.3390/membranes10050079 (PMC7281585; doi:10.3390/membranes10050079)
Supplement: Supplementary file 1 [file membranes-10-00079-s001.pdf]

Membranes

Electronic Supporting Information

Assessing the performance of thin-film nanofiltration membranes with embedded montmorillonites

Micah Belle Marie Yap Ang [1,*], Amira Beatriz Gaces Deang [2], Ruth R. Aquino [2], Blessie A. Basilia [3,2], Shu-Hsien Huang [4,1,*], Kueir-Rarn Lee [1,*], and Juin-Yih Lai [5,6,1]

[1] R&D Center for Membrane Technology and Department of Chemical Engineering, Chung Yuan Christian University, Taoyuan 32023, Taiwan; jylai@mail.ntust.edu.tw
[2] School of Chemical, Biological, and Materials Engineering and Sciences, Mapúa University, Manila 1002, Philippines; abgdeang@gmail.com (A.B.G.D.); rraquino@mapua.edu.ph (R.R.A.); basiliablessie@gmail.com (B.A.B.)
[3] Industrial Technology Development Institute, Department of Science and Technology, DOST Compound, Taguig City 1631, Philippines
[4] Department of Chemical and Materials Engineering, National Ilan University, Yilan 26047, Taiwan
[5] Applied Research Center for Thin-Film Metallic Glass, National Taiwan University of Science and Technology, Taipei 10607, Taiwan
[6] Graduate Institute of Applied Science and Technology, National Taiwan University of Science and Technology, Taipei 10607, Taiwan
* Correspondence: mbmyang@gmail.com (M.B.M.Y.A.); huangsh@niu.edu.tw (S.-H.H.); krlee@cycu.edu.tw (K.-R.L.)

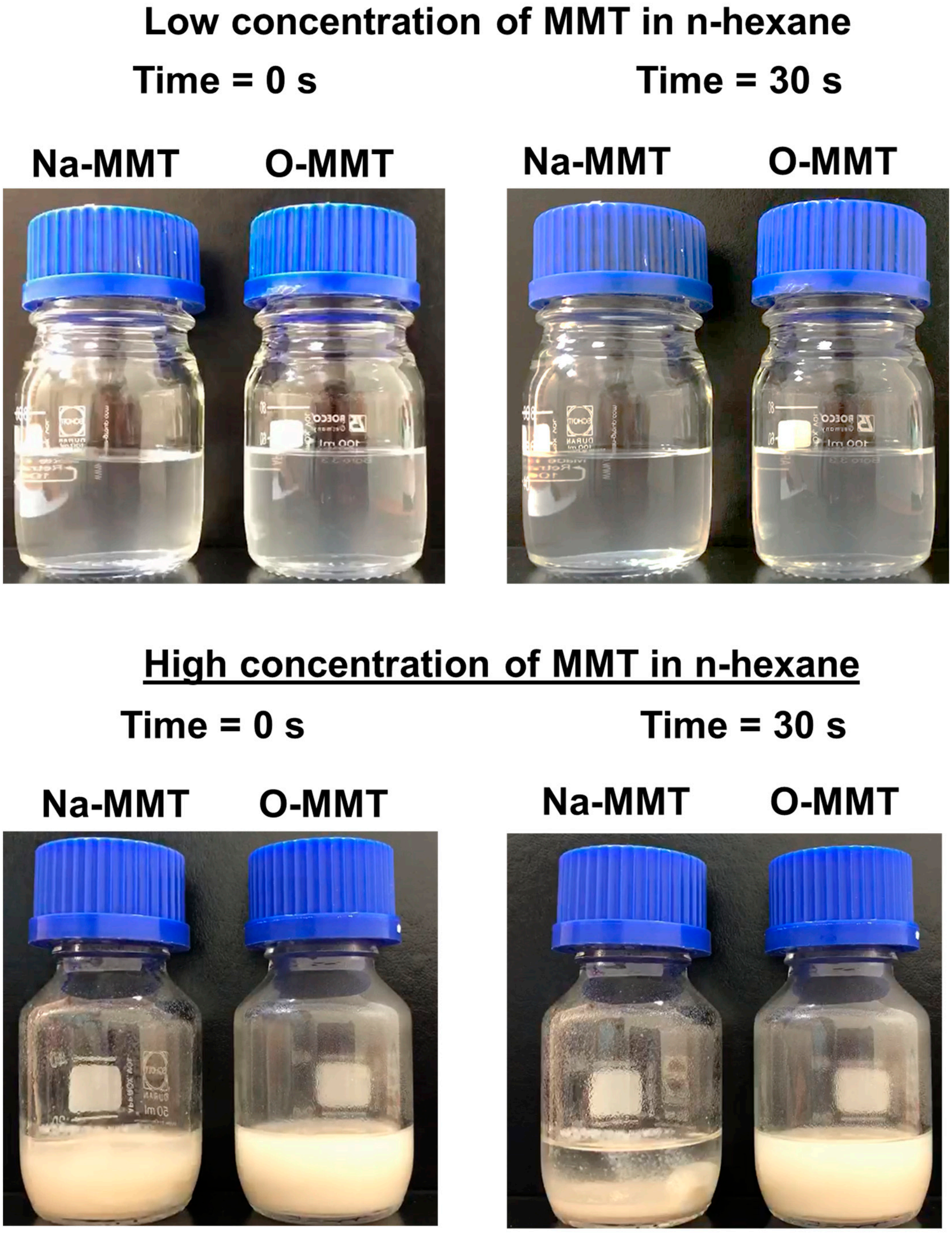

Figure S1. Dispersion of Na-MMT and O-MMT in n-hexane.

Table S1. Surface area, total pore volume and pore size of MMTs.

Particle	BET surface area ($m^2 \cdot g^{-1}$)	Langmuir surface area ($m^2 \cdot g^{-1}$)	Total pore volume [a] ($cm^3 \cdot g^{-1}$)	Pore size [b] (Å)
Na-MMT	40.12	62.43	0.136327	129.23
O-MMT	5.05	8.51	0.058832	205.34

[a] Barett-Joyner-Halenda (BJH) desorption cumulative volume of pores

[b] BJH desorption average pore diameter.
